# mSphere of Influence: an Army Marching on Its Stomach—Malaria Parasites Sense and Subvert Host Nutrition

**DOI:** 10.1128/mSphere.00210-21

**Published:** 2021-04-14

**Authors:** Clare R. Harding

**Affiliations:** a Wellcome Centre for Integrative Parasitology, Institute of Infection, Immunity and Inflammation, University of Glasgow, Glasgow, United Kingdom

**Keywords:** apicomplexan parasites, immunity, malaria, nutrition

## Abstract

Clare Harding works on the metal biology of the parasite Toxoplasma gondii. In this mSphere of Influence article, she reflects on how two papers from the laboratory of Maria Mota, “Host-mediated regulation of superinfection in malaria” by Portugal et al. (S. Portugal, C. Carret, M. Recker, A. E. Armitage, et al., Nat Med 17:732–737, 2011, https://doi.org/10.1038/nm.2368) and “Nutrient sensing modulates malaria parasite virulence” by Mancio-Silva et al. (L. Mancio-Silva, K. Slavic, M. T. Grilo Ruivo, A. R. Grosso, et al., Nature 547:213–216, 2017, https://doi.org/10.1038/nature23009), made an impact on her understanding of host-pathogen interactions by examining the complex interplay between parasites and their hosts’ nutritional status.

## COMMENTARY

Early in our careers as scientists, we are frequently presented with a simplistic narrative: a pathogen invades, and our immune system fights back. Both parties use the various weapons at their disposal—toxins or specialized killer cells—and, in the end, one army is victorious. However, this story can miss many of the most interesting interactions between the host and the parasite. Armies march on their stomachs, and the same is true for invading microbes: pathogens must acquire sustenance from their hosts to ensure replication and onward transmission. However, both the host and the pathogen are capable of manipulating the availability of nutrients to their advantage. The host can sequester or redistribute nutrients away from the invaders, and pathogens have developed intricate mechanisms to sense and subvert these nutritional changes. The papers I have chosen here reflect some of the complexity of these interactions. Their context is the deadly infectious disease malaria, a major cause of death worldwide, especially of children. Understanding how the malaria parasite acquires nutrients opens the possibility of breaking these supply lines and starving the parasite, preventing its transmission to new hosts.

In the first of these papers, the authors start with a question: why do young, only semi-immune children rarely present with two *Plasmodium* infections at the same time? Using a mouse model of malaria, Portugal and colleagues (1) found that the presence of an occupying force of parasites in the blood blocked a new *Plasmodium* infection from gaining a foothold. This was due to the production of a hormone, hepcidin, which prevented the new malaria infection from establishing its base in the liver. Although exactly how hepcidin is induced remains unknown, it is a powerful force, mediating the systemic redistribution of iron across the organism, resulting in a depletion of iron in the liver and its accumulation in macrophages, out of reach of the new invaders. The interlopers, now starved of iron, are unable to fund the huge expansion in numbers required to establish infection, while the occupying blood stage parasites can replicate happily, obtaining iron from other sources. The relevance of this pathway in humans remains under debate, in part due to the complexity of interactions between inflammation, adaptive immunity, and hepcidin on the host. However, this study uses a number of knockout mouse strains to provide clear evidence that nutrient sequestration prevents sequential infections in mice. By altering the availability of iron, the parasite acts to block superinfection, protecting its host from the often lethal effects of multiple infections and helping to ensure its own transmission.

In the second work, Mancio-Silva et al. (2) address another peculiarity of malaria infection: the observation that refeeding people after famine can be associated with malaria relapse (3). The authors, again using mouse models, demonstrate that *Plasmodium* parasites can sense their host’s nutritional status and temper their replication, growing more slowly when host nutrients are scarce, such as in calorie-restricted mice. They follow this observation with an elegant kinome screen to identify the regulatory pathway behind the parasite’s adaption to host starvation and discover a new kinase, named KIN. They follow this kinase and determine more of the pathway responsible for sensing the nutritional status (such as the availability of glucose) of the host. I like how this study paints the typically antagonistic relationship between the host and parasite in a new, more synergistic light. It is not in the parasite’s best interests to replicate too quickly under these stressful conditions since that may kill the host. Instead, the parasite responds by sensing its environment and slowing down, hoping to survive the downturn and transmit to a more amenable battleground.

These papers highlight that in many cases it is against the parasite’s best interests to kill the host too quickly. Instead, the parasite senses and responds to environmental conditions, adjusting both its own behavior and the host status to ensure continued survival and eventual transmission.

The work described here, and in other studies from the Mota group, have deeply affected my view of the interactions between the parasite and the host. Although pathogens must outrun host immunity, often causing damage to the host when they do, they also depend on their hosts in a number of complex ways. I find the interface between pathogen growth, nutrient acquisition, and the status of the host fascinating. The papers here touch on only a few aspects of nutrient sensing. For a recent account of *Plasmodium*, see the excellent review by Kumar et al. (4). However, what makes this work stand out to me is that the observation of these basic processes (e.g., slow growth in the absence of nutrients) are followed up by detailed genetic identification and characterization of the mechanisms involved. This work, which stretches from the molecular details to assessing whole-organism responses, has inspired me to look deeper at how both host processes, and the parasite, work to reshape the battlefield to their advantage. It has pushed me to think about the complex web of links between the host and pathogens which cannot be limited to a simple adversarial metaphor. But by examining these relationships in detail, we can gain insights both into the pathogens which surround us and about how our own cells and bodies function in health and disease.
